# 3D printing of hollow geometries using blocking liquid substitution stereolithography

**DOI:** 10.1038/s41598-022-26684-z

**Published:** 2023-01-09

**Authors:** Aftab A. Bhanvadia, Richard T. Farley, Youngwook Noh, Toshikazu Nishida

**Affiliations:** 1 Department of Electrical and Computer Engineering, University of Florida, Gainesville, FL 32611 USA; 2 Nanoptics, Inc., 3014 NE 21st Way, Gainesville, FL 32609 USA

**Keywords:** Surface patterning, Design, synthesis and processing, Fluidics, Lab-on-a-chip, Polymerization mechanisms, Polymers

## Abstract

Micrometer scale arbitrary hollow geometries within a solid are needed for a variety of applications including microfluidics, thermal management and metamaterials. A major challenge to 3D printing hollow geometries using stereolithography is the ability to retain empty spaces in between the solidified regions. In order to prevent unwanted polymerization of the trapped resin in the hollow spaces—known as print-through—significant constraints are generally imposed on the primary process parameters such as resin formulation, exposure conditions and layer thickness. Here, we report on a stereolithography process which substitutes the trapped resin with a UV blocking liquid to mitigate print-through. We investigate the mechanism of the developed process and determine guidelines for the formulation of the blocking liquid. The reported method decouples the relationship between the primary process parameters and their effect on print-through. Without having to optimize the primary process parameters to reduce print-through, hollow heights that exceed the limits of conventional stereolithography can be realized. We demonstrate fabrication of a variety of complex hollow geometries with cross-sectional features ranging from tens of micrometer to hundreds of micrometers in size. With the framework presented, this method may be employed for 3D printing functional hollow geometries for a variety of applications, and with improved freedom over the printing process (e.g. material choices, speed and resulting properties of the printed parts).

## Introduction

Miniaturization of and the ability to manufacture complex hollow geometries spanning across all three-dimensions (3D) is a key challenge. Hollow spaces (i.e. empty spaces, channels, cavities or voids) are necessary for a variety of device applications, for example, microfluidics (e.g. mixing and organ-on-chip)^1–3^, metamaterials (e.g. acoustic^4^, optical^5^ and mechanical^6^), circuit cooling^7^, chemical synthesis^8^ and 3D printed circuit components^9,10^. To realize hollow spaces, various microfabrication techniques can be utilized such as soft-lithography^11–14^, sacrificial templates^15–19^, micromilling^20^ and lasers^21,22^. However, these methods are slow because of the need to make photomasks and molds, or they require multiple steps that increase process integration complexities (e.g. stacking, alignment, bonding or template removal). Alternatively, arbitrary hollow geometries can be directly printed using additive manufacturing (3D printing), such as stereolithography^23,24^. While stereolithography offers high resolution and scalability to large areas^25,26^, the ability to retain micrometer scale hollow features between the solidified regions is challenging because of excess polymerization caused by “print-through”^27^.

In stereolithography, print-through is the polymerization of regions beyond the resin thickness (i.e. layer thickness) that is intended to be photopolymerized. These regions include the previously solidified layers and desired hollow spaces which contain the trapped resin. While the incident ultraviolet (UV) light attenuates with distance, according to the Beer-Lambert law^26^, the cumulative exposure dose penetrating into the hollow spaces over the course of the fabrication can lead to polymerization of the trapped resin and reduce the fidelity of the hollow geometries^28^. In addition, print-through can result in a higher viscosity of the trapped resin due to partial polymerization, thus making removal of the resin challenging after the part is fabricated.

Reduction in print-through, consequently an improvement to the vertical resolution of the process, requires optimization of the primary stereolithography process parameters—resin formulation, UV exposure dose and layer thickness of the printed parts. The complex trade-offs between these parameters and their effect on print-through has been modeled by Gong et al.^28^. Gong et al.^28^ reported that the practical hollow channel height limit is ~ 3.5–5.5*D*_*p*_, where *D*_*p*_ is the characteristic penetration depth of UV light in the resin. Therefore, the primary method to reduce print-through requires increasing the light absorbance of the resin, to reduce the value of *D*_*p*_, by tailoring the concentration of photoinitiator and absorber in the monomer. However, decreasing the exposure dose or increasing the layer thickness can also reduce the total light exposure penetrating into the hollow spaces. Various methods to mitigate print-through have been reported. Grigoryan et al.^23^ used food dyes as potent absorbers to fabricate biocompatible hydrogels that contain 1-mm cylindrical channels. Gong et al.^29^ reported development of a high-resolution resin for fabrication of 18 μm × 20 μm microfluidic flow channels by selecting an optimal absorber and layer thickness to achieve the smallest channel height. Sanchez Noriega et al.^30^ demonstrated the ability to fabricate 15 μm × 15 μm valves by a combination of using a high-resolution resin, variable exposure doses for features within the same layer, and by using variable layer thicknesses throughout the fabrication of the device. Xu et al.^31^ developed an in-situ transfer technique to reduce the total exposure dose absorbed by the trapped resin. In this technique, the capping layer which encapsulates the hollow trenches is polymerized separately, then it is transferred and bonded to the part containing the trenches using a second exposure. Together, all the reported methods attempt to modulate one or more of the primary process parameters in order to reduce the cumulative exposure dose penetrating into the hollow spaces.

Solely focusing on optimizing the primary process parameters to reduce print-through imposes significant constraints on the device development lifecycle and the properties of the printed parts. For example, the choice of materials can be restricted by the practical feasibility and manufacturability of the end application (e.g. wavelength of light source, solubility limit, cost, or biocompatibility and transparency^23,32^). Usage of high absorbance resins enforces fabrication of parts with smaller layer thickness^28^—even for the non-critical device features (e.g. inlet/outlet ports of a microfluidic device)—consequently causing the total fabrication time to be increased. Mechanical properties (e.g. hardness and Young’s modulus) are highly dependent on the process parameters^26,29,33,34^, therefore obtaining desired mechanical functionalities from certain geometries within a part^35,36^ or from the bulk device while also reducing print-through can become challenging. Decreasing the photoinitiator concentration or exposure dose can cause the part to have a weaker green strength, thus requiring additional post-fabrication curing steps. Furthermore, the lateral resolution, lateral fidelity and speed of polymerization are also affected by the resin formulation and exposure dose^37^.

We report on a stereolithography process that mitigates print-through by substituting the trapped resin in the hollow spaces with a “blocking liquid”—a liquid that attenuates the UV light, is non-polymerizing, or both. The blocking liquid is designed to reduce or eliminate polymerization within the hollow spaces to improve fidelity of the hollow geometries. Here, we investigate the mechanisms of the developed process to determine the formulation of the blocking liquid and understand the process capabilities. We find that the substituted blocking liquid enables the vertical resolution of the hollow spaces to exceed the practical limits of a conventional method without having to optimize the primary process parameters for print-through reduction. Independent of the process parameters, we show the ability to fabricate complex hollow 3D geometries with cross-sectional feature sizes ranging from tens of micrometer to hundreds of micrometers. The reported method can be adapted for a variety of device applications that require high resolution structured hollow geometries.

## Results and discussion

### Blocking liquid substitution

**Figure 1:**
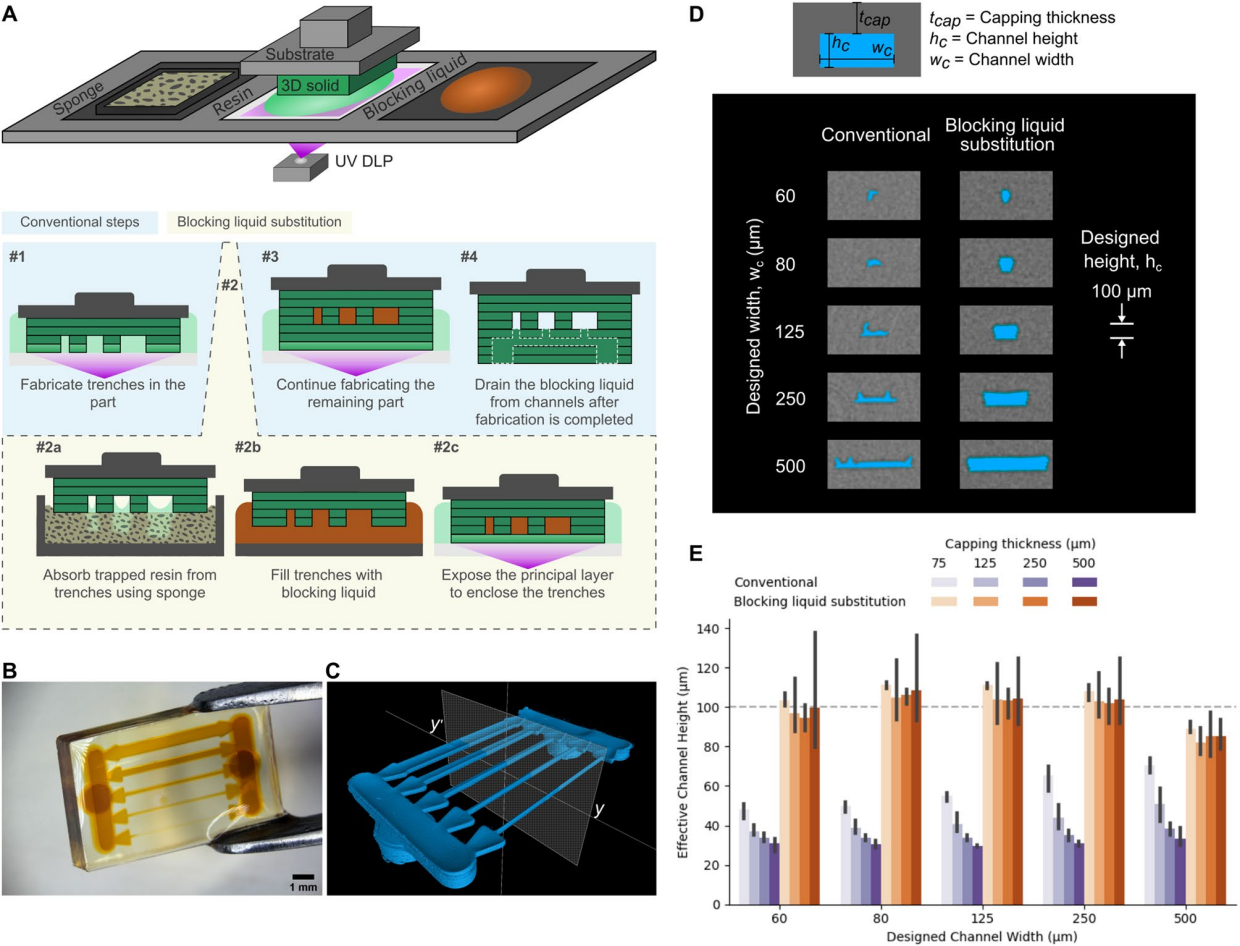
Fabrication of hollow geometries and mitigation of print-through using the blocking liquid substitution process. (**A**) Schematic of the bottom-up stereolithography system and the fabrication steps used to fabricate hollow geometries. The sponge, resin (light green) and the blocking liquid (orange) are localized on separate regions of a vat which is translatable with respect to the substrate and the part being printed (dark green). The blocking liquid substitution is selectively utilized whenever hollow geometries that are susceptible to print-through are encountered (see Supplementary Note 1), otherwise, the printing process operates in conventional mode. (**B**) Printed device (transparent) containing microfluidic channels that are filled with the substituted blocking liquid (orange). (**C**) Reconstructed 3D rendering of the hollow microfluidic channels (blue) of a printed device which was characterized using Nano-CT imaging after the substituted blocking liquid was drained out of the channels. (**D**) Comparison of the cross-sections of the hollow channels (blue) fabricated using the conventional process to those fabricated using the blocking liquid substitution process. The cross-sections were obtained using Nano-CT imaging and corresponds to the y-y’ line in (**C**). The channels had a designed height of 100 μm, and the solid capping thickness above the trenches was 250 μm. (**E**) Quantitative comparison of the hollow channels fabricated using the conventional method to the blocking liquid substitution method. The schematic of the channel cross-section in (**D**) defines geometrical parameters shown in the plot. The horizontal dashed line represents the designed channel height of 100 μm. The error bars represent standard deviation of process repeatability for n = 3 trials.

The schematic in Fig. 1A illustrates the blocking liquid substitution process to fabricate hollow geometries. The trenches in the device are initially fabricated using the conventional stereolithography process. Prior to fabrication of the principal layer—the capping layer that partially or fully encapsulates the hollow regions—the trapped resin in the trenches is substituted with a blocking liquid. The blocking liquid is introduced by first removing the trapped resin from the trenches using a sponge followed by submersing the part in a pool of the blocking liquid. The stereolithography process is resumed by printing the principal capping layer over the trenches filled with the blocking liquid. Following the exposure of the principal layer, the original resin pool can be replaced with fresh resin to mitigate contamination of the resin in the subsequent layers. After completion of the device fabrication, the substituted blocking liquid is flushed out using a solvent or drained using a vacuum. While dissolvable solid supports^38,39^ may also be used to print hollow spaces, our process avoids the need to design dissolvable resin systems, and the use of a liquid instead of a solid facilitates easier removal from complex micrometer scale hollow geometries.

A device containing the substituted blocking liquid inside an array of channels with designed widths ranging from 60 to 500 μm is shown in Fig. 1B. After removal of the blocking liquid, the internal hollow channels are characterized using the non-destructive nano-computed tomography (Nano-CT) imaging technique (see Fig. 1C). The cross-sections of the hollow channels (designed height of 100 μm) shown in Fig. 1D validates the mitigation of print-through using the blocking liquid substitution process in comparison to the conventional process.

Using the Nano-CT imaging, we measured the volume of the hollow channels to quantitatively characterize the effect of the process parameters on the fabricated effective channel height (see Methods). A higher effective channel height value corresponds to reduction in print-through, and a value closer to the designed channel height corresponds to higher fidelity. In Fig. 1E the effective channel height using the blocking liquid substitution process is compared to the conventional process as a function of the capping thickness. A larger capping thickness results in higher cumulative UV exposure dose penetrating into the hollow trenches (i.e. higher print-through). The conventional process suffers from decreasing effective channel height as the capping thickness increases (as supported by the Gong et al. model^28^), while our developed process is unaffected by the capping thickness and results in better fidelity.

### Blocking liquid interaction model

**Figure 2:**
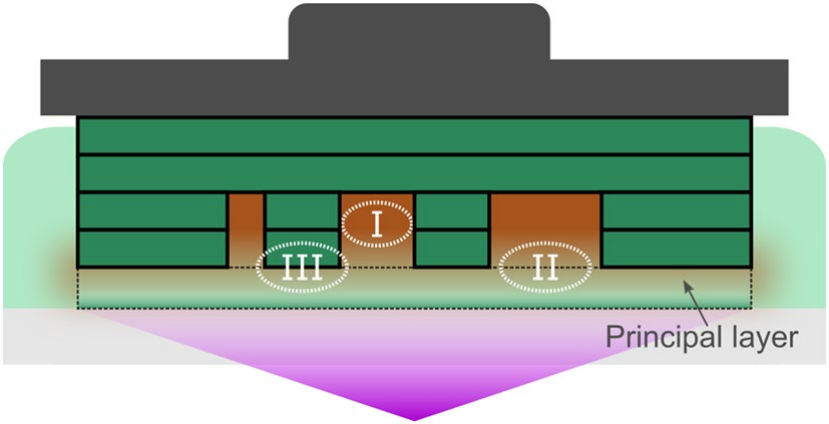
Interaction between the blocking liquid and the resin in the bottom-up stereolithography system. Schematic showing regions of interaction between the resin (light green) and the blocking liquid (orange) after substitution of the blocking liquid and during the polymerization of the principal layer. Mixing between the two liquids occurs when the part containing the blocking liquid filled trenches is brought into contact with the resin pool. The effect of liquid mixing within Regions I, II and III are discussed throughout the main text.

Here, we investigate the mechanisms of the blocking liquid substitution process and its effects on the hollow geometries and the fabricated parts. In the bottom-up stereolithography system, the blocking liquid is held in the trenches by surface tension and mixing of the resin with the blocking liquid can occur when the part is dipped into the pool of resin after the blocking liquid substitution. A schematic of three regions of liquid interactions after the blocking liquid substitution and during the polymerization of the principal layer is shown in Fig. 2. Region I is the hollow region containing the substituted blocking liquid. Region II is the boundary between the hollow region and the principal layer. Region III is the interface where the principal layer will adhere to the previously polymerized layer. All three regions can be affected by the presence and potential mixing of the blocking liquid and the resin. For instance, the diffusion of the blocking liquid into Region III decreases the adhesion strength of the principal layer to the previously polymerized layer. Therefore, the exposure dose for the principal layer—principal exposure dose—may be required to be higher than the exposure dose used for the regular layers. Likewise, the resin can diffuse into Region I (hollow region). As a consequence, the likelihood of print-through increases when the principal exposure dose is higher than the regular exposure dose. Using this model, we investigated the formulation of the blocking liquid and selection of the principal exposure dose in order to ensure high fidelity of the hollow geometries and to prevent adhesion-related defects in the principal layer.

### Formulation of blocking liquid and selection of principal exposure dose

**Figure 3:**
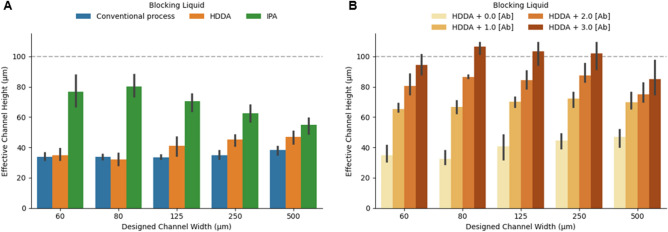
The effect of blocking liquid formulation on the effective channel height. (**A**) Comparisons of the two different blocking liquids to the conventional process. (**B**) The effect of Sudan I absorber concentration, [Ab], in HDDA blocking liquid. In (**A**) and (**B**), the designed channel height is shown as horizontal dashed lines, the principal exposure dose was four times higher than regular exposure dose, and the error bars represent standard deviation of process repeatability for n = 3 trials.

Within the hollow region (Region I), the blocking liquid functions as a substitute of the trapped resin. The requirement of the blocking liquid is that it does not polymerize or polymerizes minimally over the course of the device fabrication. This can be achieved by using a non-polymerizing liquid or a liquid that is more difficult to polymerize than the resin. We initially investigated isopropanol (IPA) and neat 1,6-hexanediol diacrylate (HDDA) monomer as the blocking liquids, and the results of each are compared to the conventional process in Fig. 3A. We observe that IPA is more effective as the blocking liquid than the neat HDDA, however, neither liquid attains effective channel heights close to the designed height. We attribute these results to the mixing of the resin into Region I during the substitution process. Furthermore, unlike IPA, the neat HDDA is more susceptible to polymerization caused by the diffusion of the photoinitiator from the resin and the higher principal exposure dose.

Although a non-polymerizing liquid can be an effective blocking liquid, using a blocking liquid that is formulated using the same components as the resin avoids introducing foreign materials into the fabrication process which can increase inhomogeneity of the principal layer or incompatibility with the resin system. For example, the resin may be diluted when using a solvent as the blocking liquid, causing the principal layer to have poor mechanical properties or weak adhesion to the previous layer (Region III). For instance, when using a HDDA based resin, we observed formation of cracks and high surface roughness in the principal layer when using IPA as the blocking liquid. Therefore, we investigated formulation of a blocking liquid which was comprised of HDDA.

To improve the effectiveness of HDDA as the blocking liquid, we investigated addition of an absorber. In Fig. 3B the effect of absorber concentration in the HDDA on the effective channel height is shown. It is found that as the absorber concentration increases, the effective channel height also increases and approaches the designed height. These results indicate that despite the mixing of the resin into the hollow regions (Region I), polymerization of the mixed liquids within the trenches (i.e. the resin and the blocking liquid) can be modulated by addition of an absorber to attenuate the UV light penetrating into the hollow regions. Since mixing of the resin into Region I is unavoidable, it is favorable to formulate a blocking liquid with a higher absorber concentration. However, the maximum limit of the absorber concentration is imposed by the effects on the inhomogeneity (e.g. mechanical properties^35^) and adhesion strength of the principal layer in Region III, and the need to prevent defects in the fabricated part. For example, when using a blocking liquid with the highest concentration of absorber investigated, we observed instances where the effective channel height was larger than the designed height (see Figs. 1E and 3B). We attribute these results to localized unpolymerized defect areas—within Region II and Region III of the principal layer—caused by the mixing of the polymerization-inhibiting blocking liquid (see Supplementary Fig. 1). These localized defects can result in poor adhesion of the principal layer and increase the likelihood of print failure.

**Figure 4:**
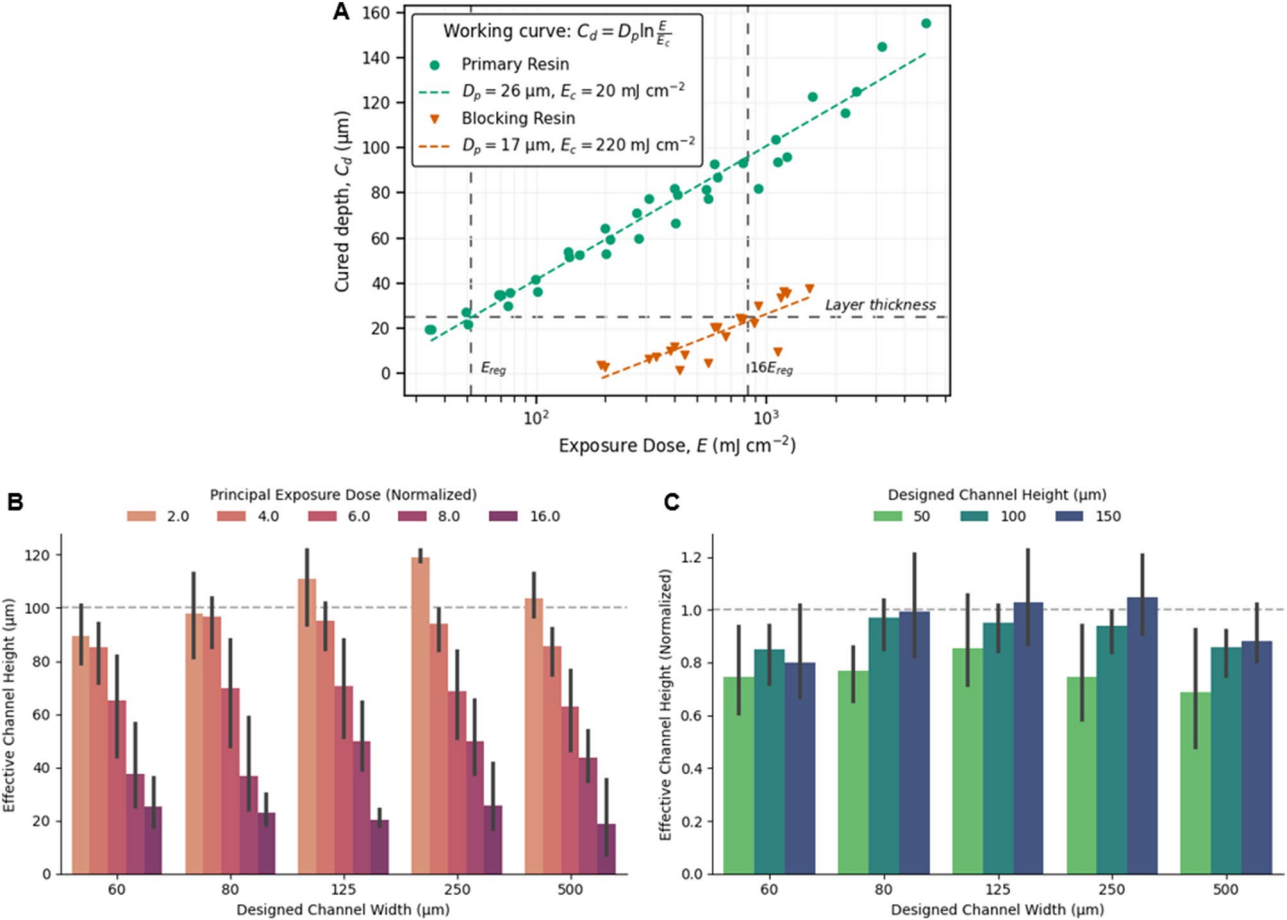
Characterization of the resin, blocking liquid and principal exposure dose, and their effects on print-through. (**A**) Photopolymerization characteristics of the resin (Primary Resin) and the blocking liquid (Blocking Resin) used for the results shown in (**B**) and (**C**). This plot shows the maximum polymerization thickness (cured depth, *C*_*d*_) that is expected for a given incident exposure dose, *E*. The trendline is fitted to the fundamental “working curve” equation of stereolithography^26^, where *D*_*p*_ is the characteristic penetration depth of light and *E*_*c*_ is the critical energy dose required for the onset of polymerization. The dashed horizontal line at 25 μm is the layer thickness and the dashed vertical line at *E*_*reg*_ is the exposure dose of the regular layers for the results shown in (**B**) and (**C**). (**B**) The effect of principal exposure dose on the effective channel height, where the exposure dose is normalized to *E*_*reg*_. (**C**) Fidelity and comparison of fabricated channels with varying designed height, where the principal exposure dose used was 4.0*E*_*reg*_. In (**B**) and (**C**) the horizontal dashed lines represent the designed channel height, and the error bars represent standard deviation of process repeatability for n = 3 trials.

Adhesion of the principal layer can be improved by intentionally adding a trace amount of photoinitiator to the blocking liquid that is highly loaded with an absorber—effectively making it a photopolymerizable resin but with very low reactivity. Although counterintuitive, we theorize that the diffusion of photoinitiator (from the blocking liquid) into Region III can minimize the dilution of the photoinitiator in the resin and improve the adhesion of the principal layer, while the high absorber concentration aids in attenuating the UV light penetrating beyond Region II and into Region I. To support this theory, in Fig. 4A we compared the photopolymerization “working curves”^26^ of the blocking liquid containing a photoinitiator (Blocking Resin) to the resin used to fabricate the solid parts in this work (Primary Resin). In stereolithography, such working curves are used to extract the UV absorbing properties and reactivity of formulated resins^26,40^. The working curves in Fig. 4A illustrate that the Blocking Resin has a higher absorption factor (lower *D*_*p*_) and lower reactivity (higher critical exposure dose requirement, *E*_*c*_) compared to the Primary Resin. These results imply that when the Blocking Resin is substituted into the hollow regions, the susceptibility of print-through will be lower in comparison to when the Primary Resin remains trapped in the hollow regions.

To confirm that the Blocking Resin can improve adhesion but also mitigate print-through, we investigated the impact of the principal exposure dose. In Fig. 4B, the effective channel height is shown as a function of the principal exposure dose normalized to the regular exposure dose (*E*_*reg*_ in Fig. 4A). We observe that when the principal exposure dose is below a critical amount, the risk of adhesion-related defects is higher because the effective channel height is greater than the designed height. As the principal exposure dose increases, the effective channel height decreases; however, even at 16× higher principal exposure dose, the effective channel height is a non-zero value. We attribute the decrease in the effective channel height to the mixing of the Primary Resin into the hollow region (Region I) and not because of the photoinitiator added to the Blocking Resin. This reasoning can be justified because the expected cure depth of the Blocking Resin is below ~ 30–40 μm at 16× higher principal exposure dose (see Fig. 4A)—a value lower than the designed channel height of 100 μm in Fig. 4B. For a given blocking liquid, appropriate selection of the principal exposure dose is necessary to balance the competing effects on the adhesion of the principal layer and the fidelity of the hollow regions below the principal layer. An optimal principal exposure dose can be determined from the required thickness of the principal layer (25 μm in our work) and the expected cure depth of the resin and the blocking liquid from the working curve characterization. For a given principal exposure dose, the actual cured depth will lie between the theoretical working curves of the resin and the blocking liquid because the principal layer contains a mixture of both liquids.

### Fabrication of complex hollow micrometer scale geometries

**Figure 5:**
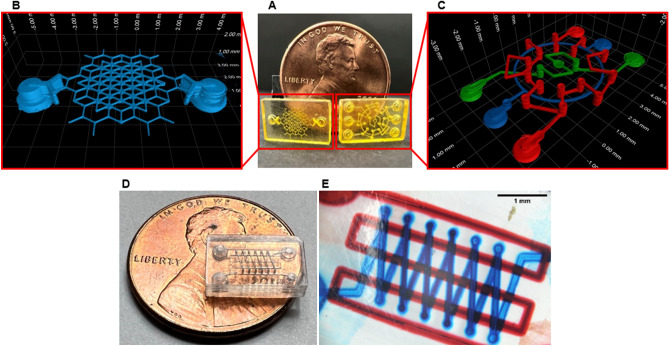
3D printed microdevices comprising of complex hollow geometries. (**A**) Picture of devices fabricated using a HDDA based resin. The hollow regions of these devices were characterized using Nano-CT imaging, for which the renderings are shown in (**B**) and (**C**). (**B**) Device comprised of channels (blue) with connected paths that form hexagonal geometric patterns (designed height of 100 μm and width of 75 μm). (**C**) Device comprised of multiple channels (designed height of 150 μm and width of 100 μm) spanning across all three-dimension of the device (multilevel channels). Each continuous channel path is colorized (red, green, blue) to illustrate that the intricate channels are separate but are interlocked. The computer-aided design (CAD) model of this device is shown in Supplementary Fig. 4. (**D**) Microscope picture of an optically transparent device comprising of multilevel channels (designed height of 250 μm and width of 125 μm) fabricated using a PEGDA based resin. (**E**) The channels of the device shown in (**D**) are filled with colored food dyes to illustrate functionality for microfluidic applications. The CAD model of the device in (**D**) and (**E**) is shown in Supplementary Fig. 5.

Using the blocking liquid substitution process, we demonstrate a variety of hollow geometries can be 3D printed using multiple types of resins and without the need to optimize the printing parameters (e.g. exposure and layer thickness) to reduce print-through. We also show that our process can exceed the hollow channel height miniaturization limit of ~ 3.5–5.5*D*_*p*_ as reported by Gong et al.^28^. In Fig. 4C the normalized effective channel height for designed heights ranging from 50 to 150 μm is shown. Using the Primary Resin with *D*_*p*_ ~ 26 μm (see Fig. 4A), channels heights as small as 50 μm (1.9*D*_*p*_) can be fabricated despite the 4× higher principal exposure dose. In Fig. 5A–C, the ability to fabricate devices with complex hollow geometries is demonstrated using another HDDA based resin that exhibits nonlinear cure depth dependence beyond a certain dose, and has an estimated *D*_*p*_ ~ 37–74 μm (see Supplementary Note 2 and Supplementary Fig. 2). The device shown in Fig. 5B illustrates an array of microfluidic channels with designed height of 100 μm (~ 1.4–2.7*D*_*p*_) arranged to form hexagonal patterns. The device shown in Fig. 5C demonstrates multiple multilevel 3D channels that are interlocked but form separate continuous paths, and have a designed cross-sectional height of 150 μm (~ 2.0–4.1*D*_*p*_). In Fig. 5D we demonstrate an optically transparent multilevel microfluidic device fabricated using a poly(ethylene glycol) diacrylate (PEGDA) based resin (see Supplementary Note 3 and Supplementary Fig. 3). In Fig. 5E the channels of the device with designed height of 250 μm (~ 4.0*D*_*p*_) are shown to be unobstructed by flushing the channels with water that was colored with food dyes. To realize transparent parts and prevent coloration (e.g. yellowing), the absorbers used in the PEGDA resin resulted in non-optimal absorption at our 385 nm UV light source. Without using our reported method, the channels of the transparent device were clogged because the PEGDA resin was highly susceptible to polymerization due to the high *D*_*p*_ ~ 62 μm and low Ec ~ 11 mJ cm^−2^. During fabrication of the devices with multilevel channels, the blocking liquid substitution process was utilized on an as-needed basis, i.e. only prior to polymerization of a layer that would pose a risk of print-through (see Supplementary Note 4). In all the presented devices, the trenches and the subsequent capping layers were fabricated with a 25 μm layer thickness and with the same exposure dose (except for the principal layer which had a higher exposure dose), and we did not utilize post-fabrication curing steps. Narrower channel heights may be achieved by fabricating the trenches with a smaller layer thickness and by formulating an appropriate blocking liquid using the framework presented in this work.

## Conclusion

The blocking liquid substitution process mitigates print-through, improves the vertical resolution and facilitates 3D printing of arbitrary micrometer scale hollow geometries. The substituted UV blocking liquid decouples the effect the resin formulation and exposure condition has on the vertical resolution of the printed hollow spaces. This decoupling can enable improved freedom over the design space of the stereolithography process such as material choices, print parameters (e.g. speed or layer thickness) and the resulting properties of the printed part (e.g. mechanical and optical). Since the blocking liquid can be formulated from the same components as the resin, adverse contamination in the process can be avoided. The substitution process can be utilized on an as-needed basis; therefore, the stereolithography system can operate in conventional mode when printing non-critical features. To further improve resolution and repeatability of our method, additional research opportunities exist. For example, use of an inhibitor in the blocking liquid can be investigated or a top-down stereolithography system can be utilized to minimize mixing during the substitution process. With the physical insights provided, our method may be adapted to further advance research and manufacturing of functional hollow geometries that are of interest in fields such as biomedical, optics, aerospace, communication, microelectronics and energy.

## Methods

### Materials

The resins investigated in this work were comprised of the following materials. Monomer: HDDA (Sartomer) and PEGDA *M*_*n*_ ~ 510 (RAHN USA Corp.). Absorbers: Sudan I (Sigma-Aldrich), Benetex OB (Mayzo) and BLS 99-2 (Mayzo). Photoinitiator: diphenyl(2,4,6-trimethylbenzoyl)phosphine oxide (TPO) (RAHN USA Corp.).

### Stereolithography system and fabrication parameters

The 3D printer was fully custom built. The control software was custom developed using Python. A Texas Instruments Digital Light Processing (DLP) projector with illumination source of 385 nm was used to pattern the UV light. The solid constraining interface (i.e. polymerization surface) used was a fluorinated ethylene propylene (FEP) film. The translatable vat contained a localized pool of resin (~ 250 μL) and blocking liquid (~ 100 μL), and a polyurethane sponge. The entire fabrication process was fully automated with the exception of when the contaminated resin had to be manually replaced with fresh resin after exposure of the principal layer.

The test device used in this study are shown in Fig. 1B,C of main text. The non-critical features (i.e. base layers with draining inlet/outlet holes) were fabricated prior to the critical features (i.e. trenches, principal layer and subsequent capping layers). The critical features were printed with 25 μm per layer thickness and exposure intensity of ~ 18 mW cm^−2^. The non-critical features were printed with 100 μm per layer thickness and exposure intensity of ~ 50 mW cm^−2^. All the test devices were fabricated using the HDDA based Primary Resin shown in Fig. 4A. The resin was comprised of TPO photoinitiator, and absorbers (Sudan I and Benetex OB) with total concentrations below 0.5% wt/wt. After filling the trenches with the blocking liquid, the part was brought in contact with the pool of resin at velocity of 100 μm s^−1^. After fabrication, the parts were sonicated in IPA for ~ 5 min followed by connecting the inlet/outlet holes of the part to vacuum to drain the blocking liquid for ~ 10 to 20 min. The parts were air dried for at least 24 h prior to analysis using the Nano-CT. Unless noted, the capping thickness was 250 μm for all discussed results in the main text.

### Characterization using Nano-CT

Fabricated parts were characterized using GE v|tome|x m 240 Nano-CT. The parts were scanned in transmission mode, with a tungsten target for the X-ray source of 80 kV and 230 μA, and with a magnification that resulted in a voxel size of 6.77 μm. After reconstruction of the scanned devices, the hollow regions of interest were segmented from the solid regions for analysis.

### Measurement of effective channel height

The reported effective channel height, *h*_*c_eff*_, in the main text is the average height of the hollow regions along a designed channel length, *L*_*c*_ = 5 mm. The effective channel height is given by the equation $h_{c\_eff} = \frac{{V_{measured} }}{{W_{c\_ref} L_{c} }}$, where $W_{c\_ref} = \frac{{V_{ref\_measured} }}{{h_{c} L_{c} }}$ is the effective channel width of a reference channel without a capping layer (i.e. *t*_*cap*_ = 0). The variable *V*_*measured*_ is the measured volume of the channel of interest, *V*_*ref_measured*_ is the measured volume of a reference channel without a capping layer, and *h*_*c*_ is the designed channel height for the channel of interest.

### Characterization of the “working curve”

The working curves of the resins shown in Fig. 4A of main text, Supplementary Fig. 2 and Supplementary Fig. 3 were characterized using the frontal photopolymerization method^40,41^. In summary, the resin was polymerized using a 1 mm square UV pattern onto a silanized glass substrate. Each pattern was sequentially exposed to form a matrix of exposure times and exposure intensities that corresponds to different exposure doses. Uncured resin from the glass surface was rinsed using IPA. The polymerized samples were then coated with a thin layer (< 100 nm) of aluminum using thermal evaporation. The cured thicknesses of polymer squares were measured using an optical profilometer. The glass substrate was silanized with 3-(trimethoxysilyl)propyl methacrylate (Gelest) using vapor phase deposition in a desiccator followed by baking the glass in an oven at 120ºC for 20 min.

### Supplementary Information

Supplementary Information.

## Data Availability

All data that support the findings of this study are available in the main text or the supplementary materials. Additional data related to this paper may be requested from the corresponding authors upon reasonable request.

## References

[1] Whitesides GM (2006). The origins and the future of microfluidics. Nature.

[2] Nguyen N-T, Wu Z (2005). Micromixers—A review. J. Micromech. Microeng..

[3] Zhang B, Korolj A, Lai BFL, Radisic M (2018). Advances in organ-on-a-chip engineering. Nat. Rev. Mater..

[4] Laureti S (2020). Trapped air metamaterial concept for ultrasonic sub-wavelength imaging in water. Sci. Rep..

[5] Zhou F (2011). Hiding a realistic object using a broadband terahertz invisibility cloak. Sci. Rep..

[6] Zheng X (2014). Ultralight, ultrastiff mechanical metamaterials. Science.

[7] Wang S, Yin Y, Hu C, Rezai P (2018). 3D integrated circuit cooling with microfluidics. Micromachines.

[8] Liu Y, Jiang X (2017). Why microfluidics? Merits and trends in chemical synthesis. Lab Chip.

[9] Wu S-Y, Yang C, Hsu W, Lin L (2015). 3D-printed microelectronics for integrated circuitry and passive wireless sensors. Microsyst. Nanoeng..

[10] Lazarus N, Bedair SS, Smith GL (2019). Creating 3D printed magnetic devices with ferrofluids and liquid metals. Addit. Manuf..

[11] Xia Y, Whitesides GM (1998). Soft lithography. Annu. Rev. Mater. Sci..

[12] Unger MA, Chou H-P, Thorsen T, Scherer A, Quake SR (2000). Monolithic microfabricated valves and pumps by multilayer soft lithography. Science.

[13] Zhang M, Wu J, Wang L, Xiao K, Wen W (2010). A simple method for fabricating multi-layer PDMS structures for 3D microfluidic chips. Lab Chip.

[14] Luan, H. *et al.* Complex 3D microfluidic architectures formed by mechanically guided compressive buckling. *Sci. Adv.* **7**, eabj3686 (2021). 10.1126/sciadv.abj3686 PMC8528415 34669471

[15] Helmer D (2017). Suspended liquid subtractive lithography: One-step generation of 3D channel geometries in viscous curable polymer matrices. Sci. Rep..

[16] Saggiomo V, Velders AH (2015). Simple 3D printed scaffold-removal method for the fabrication of intricate microfluidic devices. Adv. Sci..

[17] Kotz F (2019). Fabrication of arbitrary three-dimensional suspended hollow microstructures in transparent fused silica glass. Nat. Commun..

[18] Therriault D, Shepherd RF, White SR, Lewis JA (2005). Fugitive inks for direct-write assembly of three-dimensional microvascular networks. Adv. Mater..

[19] Patrick JF (2017). Robust sacrificial polymer templates for 3D interconnected microvasculature in fiber-reinforced composites. Compos. A Appl. Sci. Manuf..

[20] Guckenberger DJ, de Groot TE, Wan AMD, Beebe DJ, Young EWK (2015). Micromilling: A method for ultra-rapid prototyping of plastic microfluidic devices. Lab Chip.

[21] Xu B-B (2013). Fabrication and multifunction integration of microfluidic chips by femtosecond laser direct writing. Lab Chip.

[22] Waddell, E. A. Laser Ablation as a Fabrication Technique for Microfluidic Devices. in *Microfluidic Techniques: Reviews and Protocols* (ed. Minteer, S. D.) 27–38 (Humana Press, 2006). 10.1385/1-59259-997-4:27. 10.1385/1-59259-997-4:27 16508063

[23] Grigoryan B (2019). Multivascular networks and functional intravascular topologies within biocompatible hydrogels. Science.

[24] Au KA, Lee W, Folch A (2014). Mail-order microfluidics: evaluation of stereolithography for the production of microfluidic devices. Lab Chip.

[25] Vaezi M, Seitz H, Yang S (2013). A review on 3D micro-additive manufacturing technologies. Int. J. Adv. Manuf. Technol..

[26] Jacobs, P. F. & Reid, D. T. *Rapid prototyping & manufacturing: fundamentals of stereolithography*. (Society of Manufacturing Engineers in cooperation with the Computer and Automated Systems Association of SME, 1992).

[27] Shankar Limaye A, Rosen DW (2006). Compensation zone approach to avoid print-through errors in mask projection stereolithography builds. Rapid Prototyp. J..

[28] Gong H, Beauchamp M, Perry S, Woolley AT, Nordin GP (2015). Optical approach to resin formulation for 3D printed microfluidics. RSC Adv..

[29] Gong H, Bickham BP, Woolley AT, Nordin GP (2017). Custom 3D printer and resin for 18 μm × 20 μm microfluidic flow channels. Lab Chip.

[30] Sanchez Noriega JL (2021). Spatially and optically tailored 3D printing for highly miniaturized and integrated microfluidics. Nat. Commun..

[31] Xu Y (2022). In-situ transfer vat photopolymerization for transparent microfluidic device fabrication. Nat. Commun..

[32] Kuo AP (2019). High-precision stereolithography of biomicrofluidic devices. Adv. Mater. Technol..

[33] Yang Y, Li L, Zhao J (2019). Mechanical property modeling of photosensitive liquid resin in stereolithography additive manufacturing: Bridging degree of cure with tensile strength and hardness. Mater. Des..

[34] Chockalingam K, Jawahar N, Chandrasekhar U (2006). Influence of layer thickness on mechanical properties in stereolithography. Rapid Prototyp. Journal.

[35] Zhao Z (2017). Origami by frontal photopolymerization. Sci. Adv..

[36] Ji Q (2021). 4D Thermomechanical metamaterials for soft microrobotics. Commun. Mater..

[37] Bhanvadia AA, Farley RT, Noh Y, Nishida T (2021). High-resolution stereolithography using a static liquid constrained interface. Commun. Mater..

[38] Jin J, Chen Y (2017). Highly removable water support for stereolithography. J. Manuf. Process..

[39] Xu Z (2021). Vat photopolymerization of fly-like, complex micro-architectures with dissolvable supports. Addit. Manuf..

[40] Bennett J (2017). Measuring UV curing parameters of commercial photopolymers used in additive manufacturing. Addit. Manuf..

[41] Vitale A, Hennessy MG, Matar OK, Cabral JT (2015). A unified approach for patterning via frontal photopolymerization. Adv. Mater..

