# Supplementary material for: 3D printing of hollow geometries using blocking liquid substitution stereolithography
Source: Sci Rep. 2023 Jan 9;13:434. doi: 10.1038/s41598-022-26684-z (PMC9829859; doi:10.1038/s41598-022-26684-z)
Supplement: Supplementary file 1 — Supplementary Information. [file 41598_2022_26684_MOESM1_ESM.pdf]

Supplementary Information

3D printing of hollow geometries using blocking liquid substitution stereolithography

Authors: Aftab A. Bhanvadia[1], Richard T. Farley[2], Youngwook Noh[2], Toshikazu Nishida[1*]

[1]Department of Electrical and Computer Engineering Department, University of Florida, Gainesville, Florida 32611, USA.

[2]Nanoptics, Inc., 3014 NE 21st Way, Gainesville, FL 32609, USA.

*Correspondence to: E-mail: nishida@ufl.edu (T.N.)

This PDF document includes:

- Supplementary Notes
- Supplementary Figs. 1 to 5
- Supplementary References

Supplementary Notes

Supplementary Note 1: Selective utilization of the blocking liquid substitution

The blocking liquid substitution process can be used on an as-needed basis. The blocking liquid is substituted into the hollow spaces prior to polymerization of layers that would pose a risk of print-through. Otherwise, the fabrication process can continue to operate in conventional mode. See **Supplementary Note 4** for additional details on how the reported method can be utilized selectively for fabrication of multilevel hollow geometries. In a fully automated fabrication system, the pre-fabrication process planning can utilize the Gong et al. model [28] to determine when the blocking liquid substitution should be utilized based on the characteristics of the resin, the layer thickness and exposure condition used for the fabrication process.

Supplementary Note 2: Another HDDA resin

The devices in **Fig. 5A–C** of main text were fabricated using the Secondary Resin for which the working curve is shown in **Supplementary Fig. 2**. This HDDA resin was formulated with TPO photoinitiator and Benetex OB absorber. Beyond a certain dose, the cured depth of this resin exhibits a nonlinear dependence—described as “superlogarithmic behavior” by P. Jacobs [26]. Therefore, we estimated the D_p of this resin by fitting the working curve equation within the linear regime (D_p ~37 µm), and over the entire range of exposure doses tested (D_p ~74 µm) which includes the non-linear regime. Without the blocking liquid substitution process, the channels of the devices shown in **Fig. 5A–C** were confirmed to be clogged.

Supplementary Note 3: Fabrication of transparent device using PEGDA

The device shown in **Fig. 5D–E** was fabricated using a PEGDA resin for which the photopolymerization working curve is shown in **Supplementary Fig. 3**. This resin was formulated with TPO photoinitiator and absorbers (Benetex OB and BLS 99-2) which do not cause coloration (e.g. yellowing) within the visible spectrum. The critical features (i.e. trenches and capping layers) of the device were fabricated with a 25 μm layer thickness and an exposure intensity of ~6 mW cm^{-2}. We observed that the PEGDA resin is very susceptible to polymerization within the hollow regions because of the low E_c ~11 mJ cm^{-2}, therefore we formulated the blocking liquid using IPA and BLS 99-2 absorber. The lower viscosity of IPA compared to the PEGDA also facilitated draining of the blocking liquid with ease after the part was fabricated. Without the blocking liquid substitution process, the channels of the device shown in **Fig. 5D–E** were confirmed to be clogged.

Supplementary Note 4: Fabrication of multilevel hollow geometries

The fabricated device in **Fig. 5C** of the main text corresponds to the CAD model shown in **Supplementary Fig. 4**. This is an example of a device where the hollow geometries span across multiple levels of the device (along the vertical z-axis growth direction). During fabrication of this device, the blocking liquid substitution was utilized three times. The three white-horizontal dashed lines in **Supplementary Fig. 3B** indicate the boundaries of the principal layer facing the hollow region. The hollow regions located on different levels of the device are connected using hollow vias. The vias are formed when the capping layers above the hollow regions (principal layer and subsequent layers) do not fully encapsulate a hollow region which contains the blocking liquid. During the fabrication of the vias, the blocking liquid may diffuse into the resin, however, by formulating a blocking liquid comprising of the same components as the resin, inhomogeneity or contamination effects can be minimized.

Supplementary Figures

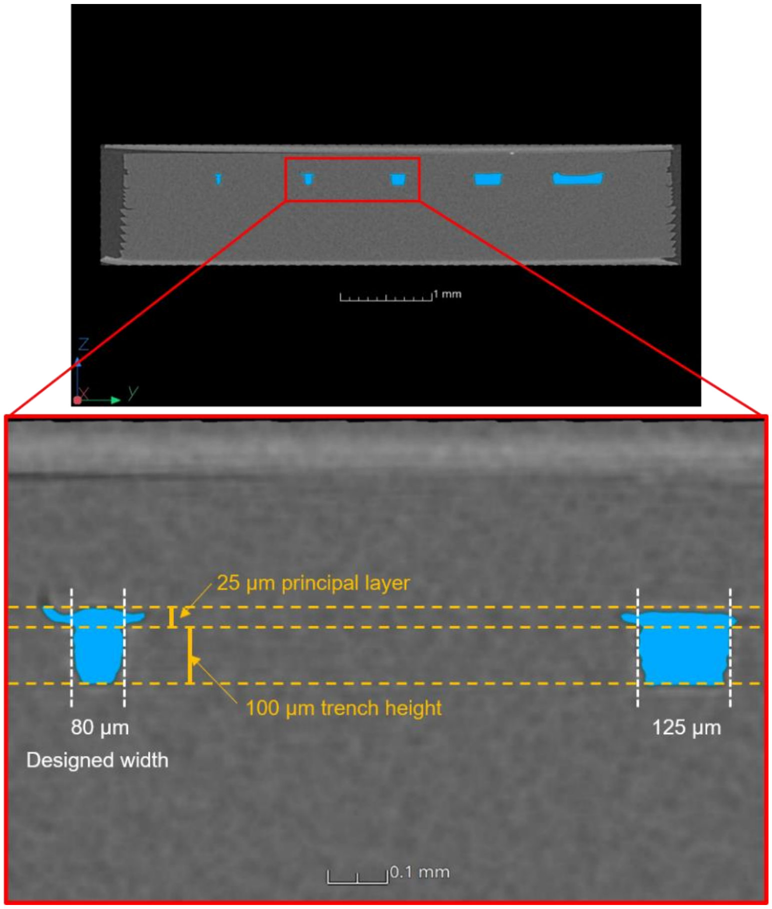

Supplementary Fig. 1. Unpolymerized regions in the principal layer. Cross-sectional view of hollow channels (blue) from a device which was fabricated using a blocking liquid comprising of HDDA + 3.0 [Ab] (see results in **Fig. 3B** of main text). The magnified image (bottom) shows that the principal layer contains unpolymerized areas that are adjacent to the trenches. These unpolymerized areas occur because of the reduced polymerization caused by the blocking liquid that has diffused from Region I into Region II and III of the principal layer (see **Fig. 2** of main text). The unpolymerized area corresponding to Region II results in hollow channel heights to be greater than the designed channel height. The unpolymerized area corresponding to Region III is an indication of poor adhesion of the principal layer.

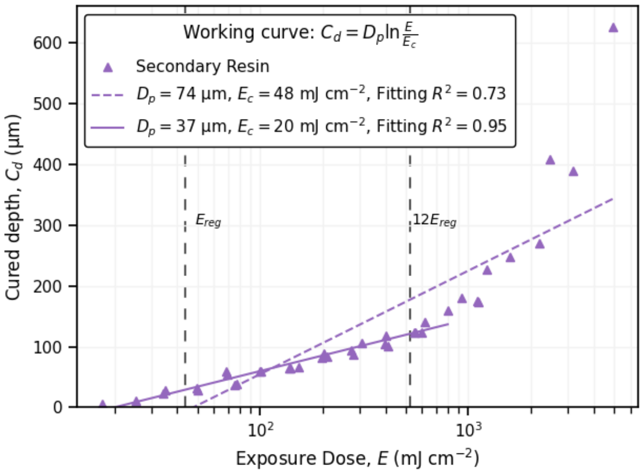

Supplementary Fig. 2. Photopolymerization working curve of another HDDA resin used in this work. This resin exhibits a superlogarithmic behavior [26] (see discussion in **Supplementary Note 2**). Two trendlines are fitted to the working curve equation in order to extract the estimated D_p value. First trendlines fits the data over the entire exposure doses tested, while the second trendline fits the data in the lower range of exposure doses where the resin behaves linearly. The vertical dashed lines at E_{reg} and $12E_{reg}$ correspond to regular exposure dose and principal exposure dose, respectively, used for the fabrication of the device in **Fig. 5C** of the main text.

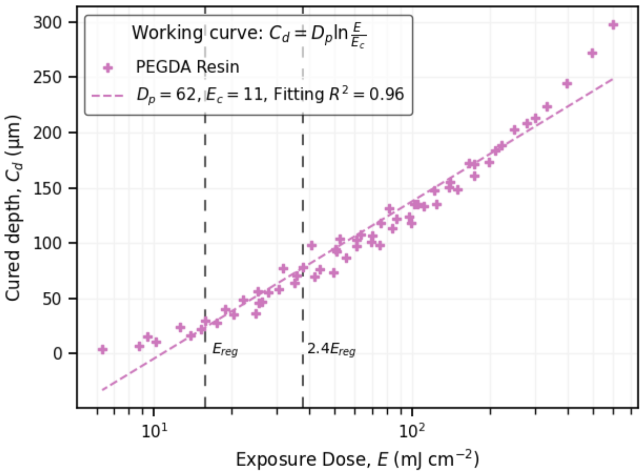

Supplementary Fig. 3. Photopolymerization working curve of a PEGDA resin investigated in this work. The vertical dashed lines at E_{reg} and $2.4E_{reg}$ correspond to regular exposure dose and principal exposure dose, respectively, used for the fabrication of the device shown in **Fig. 5D** and **Fig. 5E** of the main text.

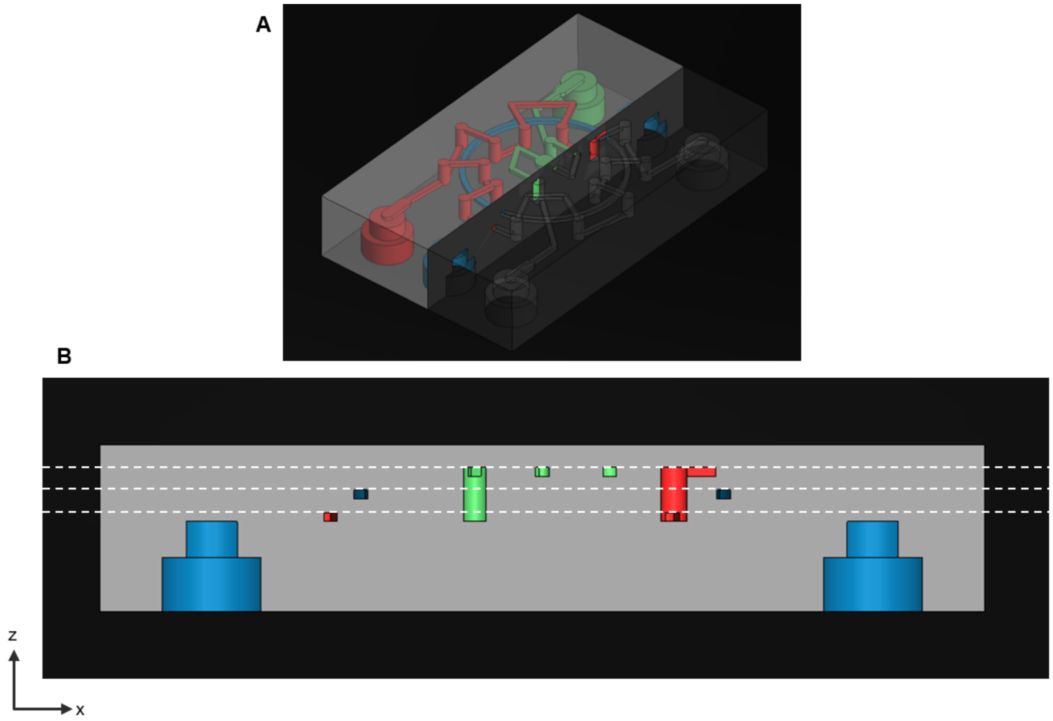

Supplementary Fig. 4. CAD model of a device with multilevel channels. This CAD model corresponds to the device shown in Fig. 5B of main text. **(A)** Perspective view of the device where the solid portion of the device is transparent in order to show the internal hollow regions that are colored red, green and blue. **(B)** One cross-sectional view of the model. The horizontal dashed lines correspond to the bottom boundary of the principal layers. Prior to polymerization of the principal layer, the hollow spaces are substituted with the blocking liquid.

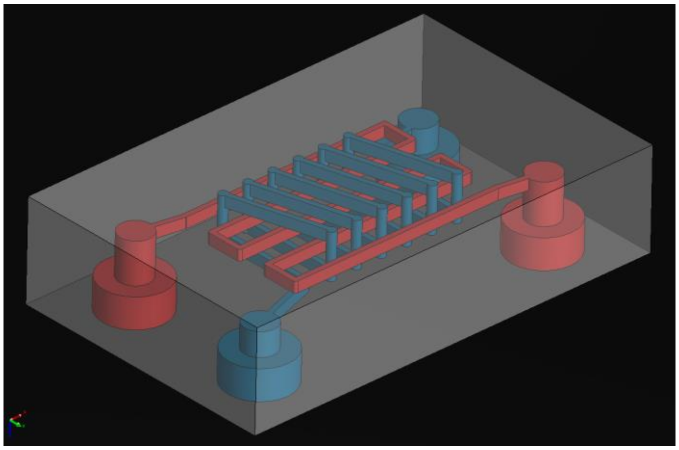

Supplementary Fig. 5. CAD model of a multilevel microfluidic device. This CAD model corresponds to the transparent PEGDA device shown in **Fig. 5D–E** of the main text. The red and blue colored regions illustrate the continuous channel paths that are separate but interlocked within the device.

Supplementary References

1. Whitesides, G. M. The origins and the future of microfluidics. *Nature* **442**, 368–373 (2006).
2. Nguyen, N.-T. & Wu, Z. Micromixers—a review. *J. Micromech. Microeng.* **15**, R1–R16 (2005).
3. Zhang, B., Korolj, A., Lai, B. F. L. & Radisic, M. Advances in organ-on-a-chip engineering. *Nat Rev Mater* **3**, 257–278 (2018).
4. Laureti, S. *et al.* Trapped air metamaterial concept for ultrasonic sub-wavelength imaging in water. *Sci Rep* **10**, 10601 (2020).
5. Zhou, F. *et al.* Hiding a Realistic Object Using a Broadband Terahertz Invisibility Cloak. *Scientific Reports* **1**, (2011).
6. Zheng, X. *et al.* Ultralight, ultrastiff mechanical metamaterials. *Science* **344**, 1373–1377 (2014).
7. Wang, S., Yin, Y., Hu, C. & Rezai, P. 3D Integrated Circuit Cooling with Microfluidics. *Micromachines* **9**, 287 (2018).
8. Liu, Y. & Jiang, X. Why microfluidics? Merits and trends in chemical synthesis. *Lab Chip* **17**, 3960–3978 (2017).
9. Wu, S.-Y., Yang, C., Hsu, W. & Lin, L. 3D-printed microelectronics for integrated circuitry and passive wireless sensors. *Microsystems & Nanoengineering* **1**, (2015).
10. Lazarus, N., Bedair, S. S. & Smith, G. L. Creating 3D printed magnetic devices with ferrofluids and liquid metals. *Additive Manufacturing* **26**, 15–21 (2019).
11. Xia, Y. & Whitesides, G. M. SOFT LITHOGRAPHY. *Annual Review of Materials Science* **28**, 153–184 (1998).
12. Unger, M. A., Chou, H.-P., Thorsen, T., Scherer, A. & Quake, S. R. Monolithic Microfabricated Valves and Pumps by Multilayer Soft Lithography. *Science* (2000) doi:10.1126/science.288.5463.113.
13. Zhang, M., Wu, J., Wang, L., Xiao, K. & Wen, W. A simple method for fabricating multi-layer PDMS structures for 3D microfluidic chips. *Lab Chip* **10**, 1199 (2010).
14. Luan, H. *et al.* Complex 3D microfluidic architectures formed by mechanically guided compressive buckling. *Sci. Adv.* **7**, eabj3686 (2021).
15. Helmer, D. *et al.* Suspended Liquid Subtractive Lithography: One-step generation of 3D channel geometries in viscous curable polymer matrices. *Sci Rep* **7**, 7387 (2017).
16. Saggiomo, V. & Velders, A. H. Simple 3D Printed Scaffold-Removal Method for the Fabrication of Intricate Microfluidic Devices. *Advanced Science* **2**, 1500125 (2015).
17. Kotz, F. *et al.* Fabrication of arbitrary three-dimensional suspended hollow microstructures in transparent fused silica glass. *Nat Commun* **10**, 1439 (2019).
18. Therriault, D., Shepherd, R. F., White, S. R. & Lewis, J. A. Fugitive Inks for Direct-Write Assembly of Three-Dimensional Microvascular Networks. *Advanced Materials* **17**, 395–399 (2005).

19. Patrick, J. F. *et al.* Robust sacrificial polymer templates for 3D interconnected microvasculature in fiber-reinforced composites. *Composites Part A: Applied Science and Manufacturing* **100**, 361–370 (2017).

20. Guckenberger, D. J., Groot, T. E. de, Wan, A. M. D., Beebe, D. J. & Young, E. W. K. Micromilling: a method for ultra-rapid prototyping of plastic microfluidic devices. *Lab Chip* **15**, 2364–2378 (2015).

21. Xu, B.-B. *et al.* Fabrication and multifunction integration of microfluidic chips by femtosecond laser direct writing. *Lab Chip* **13**, 1677 (2013).

22. Waddell, E. A. Laser Ablation as a Fabrication Technique for Microfluidic Devices. in *Microfluidic Techniques: Reviews and Protocols* (ed. Minteer, S. D.) 27–38 (Humana Press, 2006). doi:10.1385/1-59259-997-4:27.

23. Grigoryan, B. *et al.* Multivascular networks and functional intravascular topologies within biocompatible hydrogels. *Science* **364**, 458–464 (2019).

24. K. Au, A., Lee, W. & Folch, A. Mail-order microfluidics: evaluation of stereolithography for the production of microfluidic devices. *Lab on a Chip* **14**, 1294–1301 (2014).

25. Vaezi, M., Seitz, H. & Yang, S. A review on 3D micro-additive manufacturing technologies. *Int J Adv Manuf Technol* **67**, 1721–1754 (2013).

26. Jacobs, P. F. & Reid, D. T. *Rapid prototyping & manufacturing: fundamentals of stereolithography*. (Society of Manufacturing Engineers in cooperation with the Computer and Automated Systems Association of SME, 1992).

27. Shankar Limaye, A. & Rosen, D. W. Compensation zone approach to avoid print-through errors in mask projection stereolithography builds. *Rapid Prototyping Journal* **12**, 283–291 (2006).

28. Gong, H., Beauchamp, M., Perry, S., Woolley, A. T. & Nordin, G. P. Optical approach to resin formulation for 3D printed microfluidics. *RSC Adv.* **5**, 106621–106632 (2015).

29. Gong, H., Bickham, B. P., Woolley, A. T. & Nordin, G. P. Custom 3D printer and resin for 18 μm × 20 μm microfluidic flow channels. *Lab Chip* **17**, 2899–2909 (2017).

30. Sanchez Noriega, J. L. *et al.* Spatially and optically tailored 3D printing for highly miniaturized and integrated microfluidics. *Nat Commun* **12**, 5509 (2021).

31. Xu, Y. *et al.* In-situ transfer vat photopolymerization for transparent microfluidic device fabrication. *Nat Commun* **13**, 918 (2022).

32. Kuo, A. P. *et al.* High-Precision Stereolithography of Biomicrofluidic Devices. *Advanced Materials Technologies* **4**, 1800395 (2019).

33. Yang, Y., Li, L. & Zhao, J. Mechanical property modeling of photosensitive liquid resin in stereolithography additive manufacturing: Bridging degree of cure with tensile strength and hardness. *Materials & Design* **162**, 418–428 (2019).

34. Chockalingam, K., Jawahar, N. & Chandrasekhar, U. Influence of layer thickness on mechanical properties in stereolithography. *Rapid Prototyping Journal* **12**, 106–113 (2006).

35. Zhao, Z. *et al.* Origami by frontal photopolymerization. *Science Advances* **3**, e1602326 (2017).

36. Ji, Q. *et al.* 4D Thermomechanical metamaterials for soft microrobotics. *Commun Mater* **2**, 1–6 (2021).
37. Bhanvadia, A. A., Farley, R. T., Noh, Y. & Nishida, T. High-resolution stereolithography using a static liquid constrained interface. *Communications Materials* **2**, 1–7 (2021).
38. Jin, J. & Chen, Y. Highly removable water support for Stereolithography. *Journal of Manufacturing Processes* **28**, 541–549 (2017).
39. Xu, Z. *et al.* Vat photopolymerization of fly-like, complex micro-architectures with dissolvable supports. *Additive Manufacturing* **47**, 102321 (2021).
40. Bennett, J. Measuring UV curing parameters of commercial photopolymers used in additive manufacturing. *Additive Manufacturing* **18**, 203–212 (2017).
41. Vitale, A., Hennessy, M. G., Matar, O. K. & Cabral, J. T. A Unified Approach for Patterning via Frontal Photopolymerization. *Advanced Materials* **27**, 6118–6124 (2015).
